# Bioactive Phytochemicals and Antioxidant Properties of the Grains and Sprouts of Colored Wheat Genotypes

**DOI:** 10.3390/molecules23092282

**Published:** 2018-09-06

**Authors:** Oksana Sytar, Paulina Bośko, Marek Živčák, Marian Brestic, Iryna Smetanska

**Affiliations:** 1 Department of Plant Physiology, Slovak University of Agriculture in Nitra, A. Hlinku 2, 949 76 Nitra, Slovakia; marek.zivcak@uniag.sk (M.Z.); marian.brestic@uniag.sk (M.B.); 2 Department of Plant Biology, Educational and Scientific Center “Institute of Biology and Medicine”, Taras Shevchenko National University of Kyiv, Hlushkova Avenue, 2, 03127 Kyiv, Ukraine; 3 Department of Pig Breeding, Animal Nutrition and Food, West Pomeranian University of Technology in Szczecin, Klemensa Janickiego 29, 71-270 Szczecin, Poland; paulina.bosko@zut.edu.pl; 4 Plant Production and Processing, University of Applied Sciences Weihenstephan-Triesdorf, Markgrafenstr 16, 91746 Weidenbach, Germany; iryna.smetanska@hswt.de

**Keywords:** wheat, anthocyanidins, pelargonidin, cyanidin, antioxidants, antioxidant activity

## Abstract

The grains and sprouts of colored wheat genotypes (having blue, purple and yellow colored grains) contain specific anthocyanidins, such as pelargonidin and cyanidin derivatives, that produce beneficial health effects. The objective of the presented study is to compare the antioxidant capacity and contents of bioactive phytochemicals in grains and sprouts of wheat genotypes that differ in grain color. The methods α, α-diphenyl-β-picrylhydrazyl (DPPH) and 2,2′-azino-bis (3-ethylbenzothiazoline-6-sulphonic acid) (ABTS) scavenging activities, together with spectrophotometrical and high-performance thin-layer chromatography (HPTLC) methods, were used to study the presence of total phenolics, flavonoids, anthocyanins and anthocyanidins (pelargonidin, peonidin, cyanidin, delphinidin) content. It was predicted that the sprouts of all colored wheat genotypes would have significantly higher total flavonoids, total phenolics, anthocyanidin levels and antioxidant activity than the grains. The correlation results between antioxidant activity and contents of bioactive phytochemicals in grains and sprouts of colored wheat genotypes have shown a high correlation for cyanidin and pelargonidin, especially in grains, as well as quercetin in sprouts. It was found that total anthocyanin, quercetin and pelargonidin contents were significantly higher in the sprouts of the purple wheat genotypes than in the blue or yellow wheat genotypes. Delphinidin was detected at a higher level in the grains than in the sprouts of the blue wheat genotypes. Peonidin was present at very low quantities in the grains of all colored wheat genotypes. The sprouts of the purple wheat genotypes, among the colored wheat genotypes, had the highest pelargonidin, cyanidin and quercetin contents and, therefore, can be a promising source for functional food use.

## 1. Introduction

Increasing interest in the health benefits of whole wheat grain has encouraged breeders to further enhance the antioxidant contents of cereal plants [1,2,3]. The phenolic compounds are of considerable interest due to their antioxidant properties, including anthocyanins as an important subgroup of phenolic antioxidants. Phenolic compounds from purple corn are known to have anti-inflammatory, anti-carcinogenic and anti-angiogenesis properties and have great potential as food colorants [1,4,5]. Natural anthocyanin antioxidants, due to strong antioxidant capacities, have been found to mitigate lifestyle diseases, such as diabetes, obesity, hyperglycemia, hypertension and cardiovascular diseases [6,7].

Recently, the development of colored wheat genotypes among different cereal genetic resources has begun due to their high anthocyanin contents. The presence of secondary metabolites, such as tannins, polyphenols, carotenoids and anthocyanins, in wheat grains leads to a variety of grain colors. The presence of anthocyanins is determined by the *Ba* and *Pp* genes, which are responsible for the blue aleurone and purple pericarp grain colors, respectively. The yellow endosperm (*Psy* genes) of wheat grain has been shown to be regulated by the presence of carotenoids [8]. The use of pigmented wheat grains support the enrichment of total dietary fiber, phenolic acids, anthocyanins and β-glucans in the wheat-bran fraction. The presence of higher levels of secondary metabolites in the wheat-bran fraction induces higher total antioxidant activity than observed in refined flour. For example, lutein is the most abundant carotenoid in refined flour [9]. A high content of biologically active compounds, which correlates to high antioxidant activity in colored wheat genotypes, may support their use as stress-tolerant genotypes [10] or in functional food production.

The *Pp* genes acquired from the tetraploid wheat *Triticum turgidum* L. subsp. *abyssinicum* Vavilov (origin-Abyssinian region, Ethiopia) are responsible for the purple color of wheat grain, which is characterized by a high content of anthocyanins in pericarp. The cyanidins 3-glucoside, cyanidin 3-rutinoside and cyanidin 3-(6″-succinyl-glucoside) are the main anthocyanidin-3-*O*-glycosides in purple wheat grains [11,12]. The difference between purple and blue grains is due to differences in the anthocyanin composition, which is especially visible in the cross sections of kernels [11]. The dominant anthocyanins for blue aleurone wheat are delphinidin 3-rutinoside and delphinidin 3-glucoside. Cyanidin 3-glucoside and cyanidin 3-rutinoside are present in blue aleurone wheat grains, but in smaller quantities than in the purple grains [12].

The edible part of the grain of cereals is the caryopsis [3], which, in the commercially available purple wheat exhibited exceptional antioxidant capacities based on scavenging of DPPH, ABTS activities and peroxyl radicals [13]. Anthocyanin-3-*O*-glycosides-rich biscuits from purple wheat showed a high level of cyanidin 3-*O*-glucoside and exhibited high antioxidant activity [5,14]. There is currently great interest in the application of germination processes that can significantly enhance the dietary and health benefits of grains via increasing the content of bioactive phytochemicals in the sprouts [15]. Moderately hydrophobic polyphenolic fractions and hydrophilic peptidic fractions were found in wheat sprouts [16]. The presence of total phenolic compound content is correlated with high antioxidant activity [17]. The high antioxidant capacity of wheat sprouts can support their use as food supplements to act against diseases induced by free radicals [18].

The grains and sprouts of colored wheat genotypes may be potential sources of natural anthocyanin antioxidants. Research on grain composition is more available than research on sprouts. Therefore, the objective of the presented experimental work was to compare antioxidant capacity and contents of bioactive phytochemicals in grains and sprouts of wheat genotypes differing in grain color.

## 2. Results

### 2.1. Antioxidant Activity and Total Phenolic, Flavonoid and Anthocyanin Contents in Grains and Sprouts

**Figure 1:**
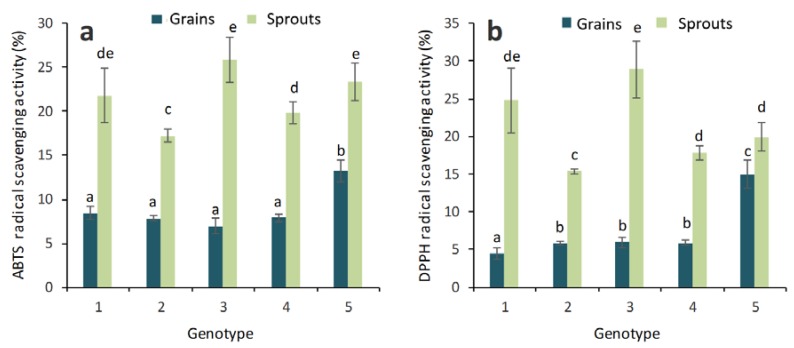
Antioxidant activity (ABTS test) (**a**) and (DPPH test) (**b**) in the grains and sprouts of colored wheat genotypes. 1—Citrus yellow, 2—KM 53-14 Blue, 3—KM 178-14 purple, 4—Skorpion Blue aleurone, 5—PS Karkulka purple. The columns represent the mean values ± S.E. for five replicates. Statistically significant differences among treatments at each time are indicated by different lowercase letters (Duncan test; *p* < 0.05).

Antioxidant activity in the grains and sprouts of colored wheat genotypes has been studied in the presented experimental work (Figure 1).

The grain sample from the genotype PS Karkulka purple was shown to have the highest antioxidant activity among the studied grains of colored wheat genotypes (ABTS and DPPH radical scavenging capacity parameters). The seedlings of the genotype PS Karkulka purple had increased antioxidant activity compared to that of the grain (18% higher) (Figure 1a,b). Three- or four-fold higher antioxidant activity was found in the seedlings of other experimental wheat genotypes.

**Figure 2:**
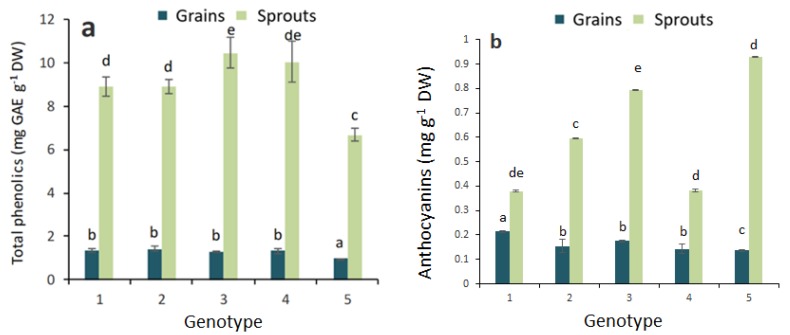
Total phenolics (**a**) and total anthocyanins (**b**) in the grains and sprouts of colored wheat genotypes. 1—Citrus yellow, 2—KM 53-14 Blue, 3—KM 178-14 purple, 4—Skorpion Blue aleurone, 5—PS Karkulka purple. The columns represent the mean values ± S.E. for five replicates. Statistically significant differences among treatments at each time are indicated by different lowercase letters (Duncan test; *p* < 0.05).

**Figure 3:**
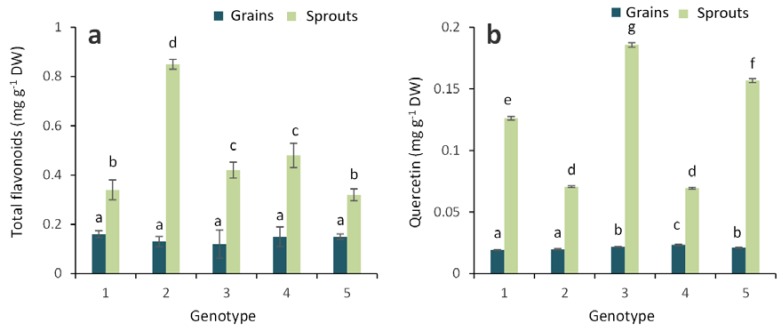
Total flavonoids (**a**) and quercetin (**b**) in the grains and sprouts of colored wheat genotypes. 1—Citrus yellow, 2—KM 53-14 Blue, 3—KM 178-14 purple, 4—Skorpion Blue aleurone, 5—PS Karkulka purple. The columns represent the mean values ± S.E. for five replicates. Statistically significant differences among treatments at each time are indicated by different lowercase letters (Duncan test; *p* < 0.05).

A significant increase in the total phenolic and flavonoid contents was observed in all studied sprouts of colored wheat genotypes (Figure 2 and Figure 3), with a similar trend of increased antioxidant activity in the sprouts of colored wheat genotypes.

There was a significantly higher anthocyanin content in the sprouts of the purple wheat genotypes than in blue or yellow wheat genotypes (Figure 2b). In addition, an increased total anthocyanin content was found in the sprouts of the yellow wheat genotype. Among the grains of the colored wheat genotypes, the lowest total anthocyanin content was measured in the Citrus yellow.

### 2.2. Anthocyanin Composition in the Grains and Sprouts of Colored Wheat Genotypes

**Figure 4:**
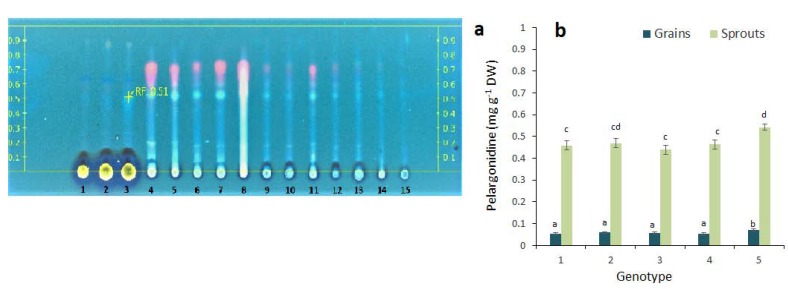
(**a**) HPTLC chromatogram of pelargonidin separation for the sprouts (tracks 4–8: track 4—Citrus yellow, track 5—KM 53-14 Blue, track 6—KM 178-14 purple, track 7—Skorpion Blue aleurone, track 8—PS Karkulka purple) and grains (tracks 9–13: track 9—Citrus yellow, track 10—KM 53-14 Blue, track 11—KM 178-14 purple, track 12—Skorpion Blue aleurone, track 13—PS Karkulka purple) extracts from colored wheat genotypes; standard mixture (tracks 1–3) (RF 0.51). (**b**) Pelargonidin contents in the grains and sprouts of colored wheat genotypes: 1—Citrus yellow, 2—KM 53-14 Blue, 3—KM 178-14 purple, 4—Skorpion Blue aleurone, and 5—PS Karkulka purple. The columns represent the mean values ± S.E. for five replicates. Statistically significant differences among treatments at each time are indicated by different lowercase letters (Duncan test; *p* < 0.05).

We found pelargonidin in all the experimental grains of colored wheat genotypes (Figure 4), and the sprouts of the colored wheat genotypes exhibited significantly higher pelargonidin contents than the grains. The highest pelargonidin content was measured in the purple genotype PS Karkulka purple.

**Figure 5:**
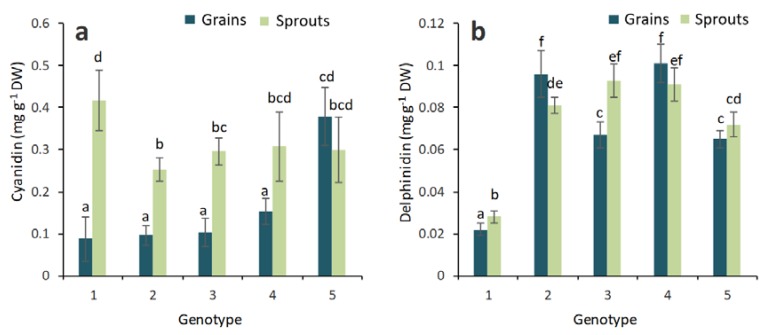
Cyanidin (**a**) and delphinidin (**b**) in the grains and sprouts of colored wheat genotypes. The columns represent the mean values ± S.E. for five replicates. 1—Citrus yellow, 2—KM 53-14 Blue, 3—KM 178-14 purple, 4—Skorpion Blue aleurone, 5—PS Karkulka purple. Statistically significant differences among treatments at each time are indicated by different lowercase letters (Duncan test; *p* < 0.05).

Our study revealed the presence of cyanidin in the blue genotypes, with higher cyanidin contents in the sprouts (Figure 5a). We suggest that a more detailed HPLC analysis of anthocyanins for further development of this research topic would be useful as well.

In our HPTLC analysis, delphinidin was identified in the grains and sprouts of the colored wheat genotypes (Figure 5b). The highest content of delphinidin was found in the blue wheat genotypes (Skorpion blue aleurone and KM 53-14 blue). The sprouts of the colored wheat genotypes did not show increased delphinidin contents. Peonidin was present at very low quantities in the grains of the colored wheat genotypes and was not identified in the sprouts (data not shown).

The correlation results between the antioxidant activity (DPPH and ABTS tests) and contents of bioactive phytochemicals in grains and sprouts of colored wheat genotypes (Table 1) has shown a high correlation for cyanidin and pelargonidin, especially in grains, as well as quercetin in sprouts. The positive correlation was also found in total phenolics, anthocyanins and flavonoids, but the level of correlation was generally moderate or low. The correlation between antioxidant activity and the delphinidin content was low in all cases.

## 3. Discussion

A significant increase in total phenolic content was observed in all studied sprouts of the colored wheat genotypes, which agrees with the similar trend of increased antioxidant activity in the sprouts of the colored wheat genotypes. There was a significantly higher anthocyanin content in the sprouts of the purple wheat genotypes than in the blue or yellow wheat genotypes. In addition, an increased total anthocyanin content was found in the sprouts of the yellow wheat genotype. Among the grains of the colored wheat genotypes, the lowest total anthocyanin content was measured in the yellow wheat genotype. This result was expected because grains of the yellow wheat genotype have yellow endosperms (*Psy* genes), which are characterized by high carotenoid levels but not high anthocyanin levels [6]. The grains of the blue and purple wheat genotypes were characterized by a higher total anthocyanin content than that in the grains of the yellow genotype. This data confirmed previous results regarding the presence of total anthocyanins in whole meal flour and in the bran of purple and blue grains [19].

Sprouts of all the colored wheat genotypes showed significant increases in total flavonoid, total phenolic contents and antioxidant activity compared to those in the grains. The novel literature shows that the sprouts of *Triticum* species may be valuable for the development of functional foods due to increased total polyphenol and free phenolic acid contents [20,21].

Anthocyanin is in the form of glycoside while anthocyanidin is known as the aglycone. The most common types of anthocyanidins are cyanidin, delphinidin, pelargonidin, peonidin, petunidin and malvidin [22]. In the HPTLC analysis we analyzed the cyanidin, delphinidin, pelargonidin and peonidin. The special interest regarding these compounds was based on their possible role in color formation of the experimental colored wheat genotypes and the health effects of specific anthocyanidins.

Anthocyanins and their aglycone forms (anthocyanidins—malvidin, cyanidin, peonidin, and delphinidin) are flavonoids which are present in notable concentrations in berries (blueberries, bilberries, cranberries, elderberries, raspberry seeds and strawberries) [23]. Consumer interest in biologically active compounds also from wheat plants that produce health effects is increasing. The presence of anthocyanidin delphinidin in the grains of blue wheat genotypes, as well as cyanidin 3-glucoside, cyanidin 3-rutinoside and succinyl glucoside in purple grains has been reported previously [12].

In the current study, we observed the presence of anthocyanidins in wheat sprouts. Specifically, anthocyanidins that are reported to be useful for prophylaxis of some diseases. Moreover, there is evidence that biologically active substances from wheat sprouts may be partially absorbed during digestion [24]. We hypothesized that grains and sprouts of specific colored wheat genotypes, in particular, can have high healthy antioxidant contents and can therefore be a promising source for functional foods.

It is known that pelargonidin chloride is present in greater amounts in the sprouts of colored wheat genotypes than in the grains. The sprouts of radish plants have been shown to have a high pelargonidin content [25]. Pink and purple pigmentation of potato sprouts (*Tuberosum* sp.) is also caused by derivatives of pelargonidin [26]. Pelargonidin chloride from plants has anti-inflammatory effects [27]. Decreasing NO production is related to the inhibition of iNOS protein and mRNA expression, which is affected by pelargonidin in a dose-dependent manner [28]. Pelargonidin chloride also has antidiabetic effects. In vitro studies have shown that insulin secretion by beta-cells increases more in the presence of a pelargonidin derivative than in the presence of a leucocyanidin derivative, which is reported to be a good anti-diabetic agent [29].

Cyanidin has been found in the grains of colored wheat genotypes and at two- to three-fold higher levels in the corresponding sprouts. Anthocyanin glucosides, namely cyanidin 3-*O*-rutinoside, cyanidin 3-*O*-glucoside, cyanidin 3-*O*-galactoside and cyanidin 3-*O*-galactopyranosyl-rhamnoside, were detected in common buckwheat [30]. Knievel et al. (2009) [12] showed that in purple grains, cyanidin 3-glucoside and cyanidin 3-rutinoside are the main anthocyanidin-3-*O*-glycosides, but their contents are lower in blue grains due to the presence of specific blue color genes (*Ba* genes) and purple pericarp color genes (*Pp* genes) [8]. Our study has demonstrated the presence of cyanidin in the grains of blue genotypes of wheat, with higher cyanidin content in their sprouts.

The anthocyanidins cyanidin and delphinidin possess high radical scavenging activity, suppress cell proliferation and increase the apoptosis of MCF7 breast cancer cells [31]. In the previous HPTLC analysis, the 3-glucosides of delphinidin, cyanidin, malvidin and peonidin, further cyanidin glycosides and respective anthocyanidins were found in powdered berry extracts [32]. In the present experiment, the highest content of delphinidin was observed in the blue wheat genotypes (Skorpion blue aleurone and KM 53-14 blue). The sprouts of the purple and yellow wheat genotypes did not show increased delphinidin contents. Delphinidin and its derivatives were also found in the sprouts of radish and buckwheat plants [25,33]. The effects of hydroxycinnamic acids on blue color expression of cyanidin derivatives have been studied, and it was shown that interactions with phenolic compounds can play important roles in color expression [34].

The study of colored wheat genotype sprouts showed a similar trend of increased total flavonoids content [35]. The presence of quercetin in wheat sprouts has been previously demonstrated [36]. It was confirmed that the nutritional promise of wheat sprouts was due to high catalase and peroxidase activity, together with a high content of organic phosphates [23]. The health effects of quercetin are well studied, especially quercetin’s ability to scavenge hydroxyl radical, peroxynitrite and other free radicals [36]. The possible use of cyanidin derivatives as nutraceuticals has also been discussed [36].

The methods used in this study did not cover the full spectrum of anthocyanins, which, in turn, provides potential for future research focused on analyzing the full profile of anthocyanidins and quercetin compounds. Moreover, the presented correlation analysis between the individual phytochemicals and the antioxidant capacity is informative for further investigations. In addition, the link between the presence of anthocyanidins and cyanidin derivatives in grains and sprouts, and their role in plant stress resistance could be interesting, as it was previously tested in salt stress experiments [37]. Thus, the colored wheat genotypes are becoming an increasingly attractive target for biological and food research.

## 4. Materials and Methods

### 4.1. Reagents and Chemicals

All anthocyanin standards were obtained as HPLC-grade chloride salts. Cyanidin (cn; 3,30,40,5,7-pentahydroxyflavylium), delphinidin (dp; 3,30,40,5,50,7-hexahydroxyflavylium) and peonidin (pn; 3,40,5,7-tetrahydroxy-30-methoxyflavylium) were provided by Cayman chemistry (Hamburg, Germany). Pelargonidin (pg; 3,40,5,7-tetrahydroxyflavylium) was provided by Sigma–Aldrich (Darmstadt, Germany). For the mobile phases, ethyl acetate and toluene (Merck, Darmstadt, Germany), as well as formic acid (Sigma–Aldrich, Darmstadt, Germany), were purchased at HPTLC grade. Double-distilled water was prepared using a Heraeus Destamat Bi-18E (Thermo Fisher Scientific, Schwerte, Germany). Methanol (gradient grade and LC-MS Chromasolv), hydrochloric acid (37% and 25%, reagent grade) and 2,2-diphenyl-1-picrylhydrazyl radical (DPPH) were ordered from Sigma–Aldrich, Darmstadt, Germany. All chemicals used for the bioassay, the HPTLC silica gel plates (60 F254, 20–10 cm) and the potassium acetate used for humidity control during plate development were purchased from Merck. The HPTLC plates were pre-washed with methanol to the upper plate edge and dried at 120 °C for 15 min on a thin-layer chromatography (TLC) plate heater (CAMAG, Muttenz, Switzerland). The plates were cooled to room temperature in a desiccator with phosphorus pentoxide (P99%, Sigma–Aldrich, Darmstadt, Germany) and temporarily stored while protected by a clean glass plate and wrapped in aluminum foil.

### 4.2. Plant Objects

**Figure 6:**
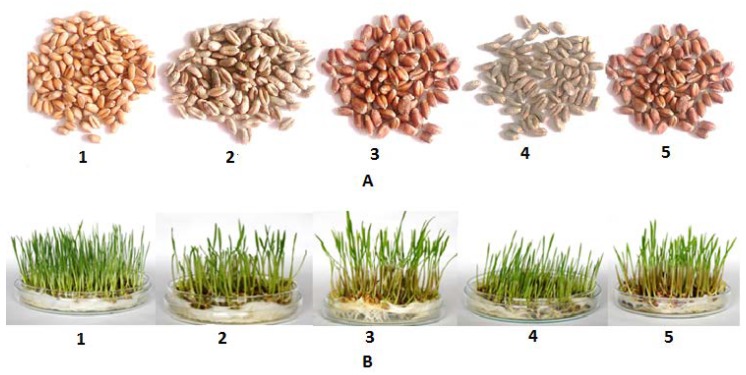
Experimental differently pigmented wheat grains (**A**) and sprouts (**B**). 1—Citrus yellow, 2—KM 53-14 Blue, 3—KM 178-14 purple, 4—Skorpion Blue aleurone, 5—PS Karkulka purple.

Seeds of wheat genotypes (*Triticum* sp.) with different pigments—Citrus yellow, KM 53-14 Blue, KM 178-14 purple, Skorpion Blue aleurone and PS Karkulka purple—were provided by the Agricultural Research Institute Kromeriz, Kromeriz, Czech Republic. The pigmented seeds were characterized based on visual pink, blue and yellow color assessment (Figure 6).

Grains were washed extensively with distilled water and sterilized with 5% sodium hypochlorite for 5 min. Then, seeds were sown in petri dishes with absorbent pads and directly watered with 3 mL of 1/4 strength Hoagland’s nutrient solution [38]. The germination process proceeded under controlled conditions in a growth chamber with the following parameters: Relative humidity of 60–70% and light/dark regime of 16/8 h at 25/20 °C. The length of germination process was 10 days. Then, 5 cm sprouts were harvested and frozen in liquid nitrogen to prevent phenolic compounds volatization. Samples were taken for analysis after the freeze-drying process was complete, where the material was ground by a flint mill (20,000× *g*, 2 min).

### 4.3. Determination of DPPH Radical Scavenging Capacity

The DPPH assay previously described [39] was used with some modifications. The stock reagent solution (1 × 10^−3^ M) was prepared by dissolving 22 mg of DPPH in 50 mL methanol. The stock solution was stored at 20 °C until use. The weight of the samples was 0.02 g, and all samples were assayed six times. The extraction was performed in two steps: First, 0.02 g of dry material was placed in an Eppendorf tube, and 1 mL of distilled water was added to the tube. The samples were heated for 15 min at 95 °C. Then, the material was centrifuged for 5 min (12,000 rpm, 25 °C). The extract was added to a new tube.

The supernatant was diluted with 1 mL of distilled water, reheated for 10 min at 95 °C and then spun again (12,000 rpm, 25 °C, 5 min). The extract was added to a new tube. The working solution (6 × 10^−5^ M) was prepared by mixing 6 mL of the stock solution with 100 mL of methanol. The optical absorbance was measured at 515 nm with a Jenway UV/Vis 6405 spectrophotometer (Jenway, Chelmsford, UK). Then, 0.1 mL of each experimental extract was mixed with 3.9 mL of the DPPH solution to react, followed by vortexing for 30 s and a further reaction time of 30 min. The absorbance was measured at 515 nm. A sample with no added extract was used as a control. The DPPH scavenging capacity was determined based on the following formula:

DPPH scavenging capacity (%) = [(A_control_ − A_sample_)/A_control_] × 100 (1)

A = absorbance at 515 nm.

### 4.4. ABTS Radical Scavenging Assay

For the ABTS assay, the method of Re et al. (1999) was adopted [40]. The stock solutions included 7 mM ABTS solution and 2.4 mM potassium persulfate solution. The working solution was prepared by mixing equal quantities of the two stock solutions and allowing them to react for 12–16 h at room temperature in the dark. The mixture was diluted by mixing 1 mL ABTS solution with 60 mL of methanol to obtain an absorbance of 0.706 ± 0.001 units at 734 nm using the spectrophotometer (Jenway 6505 UV/Vis). The ABTS solution was freshly prepared for each assay. The aqueous extraction of samples was undertaken. A total of 0.02 g of dry material was placed in an Eppendorf tube, and 1 mL of distilled water was added to the tube. The samples were heated for 15 min at 95 °C. Then, the material was centrifuged for 5 min (12,000 rpm, 25 °C). The procedure was repeated twice, with supernatants collected in the separate Eppendorf tube. Sample extracts (1 mL of each) could react with 1 mL of the ABTS solution, and the absorbance was taken at 734 nm after 7 min using the spectrophotometer. The ABTS + scavenging capacity of the extract was calculated as percentage inhibition.

ABTS radical scavenging activity (%) = [(Abs_control_– Abs_sample_)]/(Abs_control_)] × 100 (2)

where Abs_control_ is the absorbance of ABTS radical + methanol; Abs_sample_ is the absorbance of ABTS radical + sample extract.

### 4.5. Determination of Total Phenolics

The total phenolic content was estimated using the Folin–Ciocalteu reagent [41]. Twenty milligrams of freeze-dried samples in the form of powder were mixed with 500 μL of 70% methanol (HPLC-Gradient grade, Sigma–Aldrich, Darmstadt, Germany) at 70 °C for 10 min. The extracts were centrifuged for 10 min at 3500× *g*. The supernatants were collected in individual tubes. The pellets were re-extracted under the same conditions. The supernatants were combined and used to estimate the total phenolic content, and 20 μL of extract was dissolved into 2 mL of distilled water for the total phenolics analysis. Folin–Ciocalteu reagent, previously diluted ten-fold with distilled water and kept at 25 °C for 3–8 min, was used for the analysis; 200 μL of dissolved extract was mixed with 0.8 mL of sodium bicarbonate (75 g L^−1^) solution, and 1 mL of Folin–Ciocalteu reagent was added to the mixture. The mixture was left to react for 60 min at 25 °C. Samples of sprouts were taken after finishing the freeze-drying process where the material was ground by a flint mill (20,000× *g*, 2 min). The absorbance for total phenolics was detected at 765 nm with a Jenway UV/Vis 6405 spectrophotometer (Jenway, Chelmsford, UK). The results are described as gallic acid equivalents (GAE/g sample).

### 4.6. Detection of Total Flavonoid Content

The total flavonoid content was detected using the aluminum chloride colorimetric method. Samples weighing 0.1 g were used for flavonoid extraction with 7 mL of 95% ethanol for 16–18 h. Then, 0.5 mL of the extract stock solution was reacted with 1.5 mL of 95% ethanol, 0.1 mL of 10% aluminum chloride, 0.1 mL of 1 M potassium acetate, and 2.8 mL of distilled water. Aluminum chloride solution was replaced with the same amount of distilled water in the blank. After the mixture was incubated for 30 min at room temperature, the absorbance of the reaction mixture was measured at λ = 415 nm. Quercetin was used as a standard for the calibration curves. Ten milligrams of quercetin were dissolved in ethanol, and then, the solution was diluted to 25, 50 and 100 g mL^−1^. A calibration curve was created by measuring the absorbance of the dilutions at 415 nm (λ_max_ of quercetin) with a Jenway 6405 UV/Vis spectrophotometer.

### 4.7. Estimation of Anthocyanins

Total anthocyanins were estimated by a pH-differential method [42]. A known weight of samples was soaked in 3 mL of acidified methanol (1% *v*/*v* HCl) for 24 h in darkness at 4 °C with occasional shaking. Approximately 2 mL of distilled water and 4.8 mL of chloroform were mixed and added to the extract. The mixture was centrifuged for 15 min at 5000× *g*. The absorbance of the upper phase was determined at 530 and 657 nm [43]. The concentrations of the anthocyanins as mg g^−1^ dry weight of the differently treated plants was determined using the following equation:

Total Anthocyanins = [OD530 − 0.25 OD657] × TV/[DW × 1000](3)

OD = optical density; TV = total volume of the extract (mL); DW = weight of the dry leaf tissue (g). The anthocyanins content was finally expressed as mg cyanidin-3,5-diglucoside equivalents per g DW.

### 4.8. Stock Solutions and Sample Preparation for HPTLC

Anthocyanin stock solutions of 1 mg mL^−1^ each were individually prepared in a mixture of methanol and hydrochloric acid 25% (4:1, *v*/*v*). For the anthocyanin stock solutions, cyanidin chloride, pelargonidin chloride, peonidin chloride and delphinidin chloride (each 1 mg/mL) were individually dissolved in acidified methanol, and the same preparation was used for quercetin.

For sample preparation, 0.2 g of wheat DW of each sample was dissolved in 2 mL of acidified methanol (methanol and hydrochloric acid 25% mixture, 4:1, *v*/*v*). After sonication for 30 min at room temperature, the solutions were filtered through 0.45 µm cellulose filters and stored at −20 °C.

### 4.9. TLC Conditions

TLC aluminum oxide 60 F_254_ plates (10 × 10 cm, Merck, Darmstadt, Germany) were used for the TLC analysis. Standards with different volumes (1, 2, 4, 6 μL) and samples in 4–6 μL volumes were applied. The prepared stationary phase was applied using a Linomat Vapplicator (CAMAG, Muttenz, Switzerland) under the next plate’s condition with 10-mm-wide bands at 10 mm from the edge, with 10 mm from the plate bottom and a distance of 8 mm between the bands.

A mixture of chloroform/methanol/acetone/ammonia 25% (10:22:53:0.2, *v*/*v*/*v*/*v*) was used for the mobile phase. Chromatograms were prepared in glass chromatographic chambers (17.5 × 16 × 8.2 cm in size; Sigma–Aldrich, Darmstadt, Germany). The conditions for developing the chromatogram were room temperature with 9 cm over 40 min. Saturation was established for 15 min with vapor of the mobile phase. The plates were scanned after they were air-dried in the dark. The resulting spots were analyzed at 254 and 366 nm using a TLC Scanner 3 (CAMAG, Muttenz, Switzerland).

### 4.10. High-Performance Thin-Layer Chromatography

HPTLC methods for analysis of anthocyanidins in powdered, freeze-dried wheat samples were not found in the literature, except for the analysis of anthocyanidins in powdered berry samples [32]. Samples of sprouts were taken after the freeze-drying process was completed, where the material was ground by a flint mill (20,000× *g*, 2 min). Up to 18 tracks can be applied on one 20–10 cm HPTLC plate. For four-point-calibration, volumes of 2, 4, 6, and 8 µL of the standard mixture solution were applied at 20–250 ng/band, depending on the substance. Sample volumes of 2, 4 or 6 µL were used for the anthocyanidin analyses, depending on the expected anthocyanin concentration. For unknown concentrations, various volumes were applied, and the volume showing the best separation performance (not overloaded but above LOQ) was applied three-fold and used for quantitation. This volume was usually 2 or 4 µL for the given sample preparation. The plate activity was adjusted at a relative air humidity for 4 min at 25 ± 2% in the humidity control unit with a saturated potassium acetate solution (260 g/100 g water). The migration distance was 70 mm from the lower plate edge, and the migration time was 30 min. After development, the plate was automatically dried in a stream of cold air for 3 min.

For anthocyanidins separation, the plate was cut below the co-eluting anthocyanidin fraction (below solvent front) of the first solvent system using the TLC Plate Cutter (CAMAG, Muttenz, Switzerland). The upper plate part was developed with a mixture of ethyl acetate:toluene:formic acid:water (10:3:1.2:0.8; *v*/*v*/*v*/*v*) to a migration distance of 45 mm from the lower plate edge (almost to the plate top) in a twin-trough chamber (20–10 cm, CAMAG, Muttenz, Switzerland). Images of the plates were recorded with a TLC Visualizer documentation system equipped with a high-resolution 12-bit CCD digital camera (CAMAG, Muttenz, Switzerland). As the content of anthocyanidins is lower than that of anthocyanins, higher sample volumes had to be applied, which led to an overloaded separation of the anthocyanins [32]. The same sample should be applied at a low and high volume, e.g., 2 and 10 µL. This combined 2-step method was recommended by Krüger et al. (2013) [44].

The images were taken under white light illumination in the transmission mode with an exposure time of 35 ms. Spectra of the corresponding zones were used for the determination of wavelengths to measure individual anthocyanidins. A TLC Scanner 4 (CAMAG, Muttenz, Switzerland) was used for anthocyanidin detection. Densitometric evaluation was completed with a halogen tungsten lamp. The multi-wavelength scan was used to measure absorbance at 555 nm for delphinidin, at 530 nm for cyanidin and at 520 nm for pelargonidin and peonidin. The measuring scanning speed was 20 mm/s and the slit size was 6 mm–0.2 mm. The data were processed with the software winCATS, Version 1.4.7.2018 (CAMAG, Muttenz, Switzerland).

### 4.11. Statistical Analysis

Statistical analyses were performed using a two-way (genotype × sample type) analysis of variance (ANOVA) and Duncan’s multiple range test performed at *p* = 0.05 (STATISTICA 10, StatSoft, Tulsa, OK, USA). Mean values were calculated from five replicates per cultivar for each type of sample (sprouts and grains). Data are presented as mean ± standard error from five replicates (SE). The correlation analysis between the antioxidative activity results and the phytochemicals contents was analyzed using the Statistica software, providing a correlation index and statistical significance (*p* < 0.05) for each correlation.

## 5. Conclusions

The comparative study of the grains and sprouts of colored wheat genotypes showed that sprouts have higher contents of biologically active compounds of a polyphenolic nature and antioxidant activities than grains. This result indicates that wheat sprouts from colored wheat genotypes are a valuable source for further food or pharmaceutical use. The significantly high content of pelargonidin and cyanidin derivatives in the sprouts of purple wheat genotypes, together with their high antioxidant activity, makes them valid candidates for functional food production. The genetic variability of colored wheat genotypes supports the availability of specific anthocyanidins from wheat sprouts: Blue wheat genotypes contain mostly delphinidin, and purple genotypes have a high quantity of pelargonidin. Further studies are needed to qualitatively analyze colored wheat sprouts, especially HPLC analyses of flavonoids and anthocyanins in the form of glycoside compositions.

## Figures and Tables

**Table 1:** Results of the correlation between antioxidant activity and contents of bioactive phytochemicals in grains and sprouts of colored wheat genotypes.

Sample	Antiox Activ.	Total Phenolics	Total Anthocyan	Total Flavonoids	Quercetin	Pelargonidin	Cyanidin	Delphinidin
**Grains**	**ABTS**	0.51 *	0.29	0.43 *	0.08	0.68 *	0.96 *	0.04
	**DPPH**	0.62 *	0.38	0.22	0.20	0.59 *	0.96 *	0.09
**Sprouts**	**ABTS**	0.34	0.48	0.16	0.69 *	0.55 *	0.63 *	0.22
	**DPPH**	0.48	0.21	0.14	0.67 *	0.26	0.60 *	0.02

* Correlations are significant at *p* < 0.05000.
